# The association between cigarette smoking and obstructive sleep apnea

**DOI:** 10.18332/tid/105893

**Published:** 2019-04-05

**Authors:** Wen-Yu Hsu, Nan-Ying Chiu, Cheng-Chen Chang, Ting-Gang Chang, Hsien-Yuan Lane

**Affiliations:** 1 Graduate Institute of Clinical Medical Science, China Medical University, Taichung, Taiwan; 2 Department of Psychiatry, Changhua Christian Hospital, Changhua, Taiwan; 3 School of Medicine, Chung Shan Medical University, Taichung, Taiwan; 4 Department of Psychiatry & Brain Disease Research Center, China Medical University Hospital, Taichung, Taiwan; 5 Department of Psychology, College of Medical and Health Science, Asia University, Taichung, Taiwan

**Keywords:** cigarette smoking, obstructive sleep apnea, apnea–hypopnea index, risk factor

## Abstract

**INTRODUCTION:**

Obstructive sleep apnea (OSA) is a serious sleep disorder characterized by repetitive episodes of paused or shallow breathing during sleep. Patients with OSA often have excessive daytime sleepiness. The role of cigarette smoking in OSA remains controversial. The aim of this study was to examine the relationship between cigarette smoking and OSA.

**METHODS:**

In this retrospective chart review, we reviewed 18-month sleep laboratory charts in central Taiwan. We collected data regarding sleep, current cigarette smoking status, sex, age, body mass index (BMI), neck circumference, Epworth Sleepiness Scale score, and polysomnographic sleep parameters. In total, 733 subjects were recruited; among these, 151 were smokers and 582 were non-smokers.

**RESULTS:**

Smokers had significantly higher apnea–hypopnea index (p<0.001) for non-rapid eye movement sleep stage, higher apnea–hypopnea index (p<0.001) for total sleep time, and higher snore frequency (p<0.001) in t-test analysis. They also demonstrated higher Epworth Sleepiness Scale scores, shorter sleep times, lower percentage of slow-wave (deep) sleep, and longer snore times. However, no significant association was found between cigarette smoking and OSA after adjusting for sex, age, and BMI (OR=1.02, 95% CI: 0.66–1.57).

**CONCLUSIONS:**

We did not find any significant association between cigarette smoking and OSA after adjusting for age, sex, and BMI. Further well-designed prospective controlled cohort studies might clarify the relationship between cigarette smoking and OSA.

## ABBREVIATIONS

OSA: obstructive sleep apnea
OR: odds ratio
CI: confidence interval
BMI: body mass index
AHI: apnea–hypopnea index
PSG: polysomnographic

## INTRODUCTION

Obstructive sleep apnea (OSA) is characterized by complete or partial airway obstruction during sleep, which can cause substantial physiological disturbance and various clinical impacts. Several diseases, including diabetes, hypertension, myocardial infarction, and gastroesophageal reflux disease, are associated with OSA^1-4^. OSA causes frequent brief arousals and poor sleep quality. Consequently, patients with OSA frequently experience excessive daytime sleepiness^5^. As the disorder progresses, the associated sleep drive becomes increasingly irresistible and endangers patients. In addition, lack of sleep may induce cognitive dysfunction, poor concentration, impaired memory and judgment, irritability, and depression^6,7^. Thus, several diseases with potentially lethal severity are associated with OSA, however, suggesting OSA as the common link requires considerable attention. The widely accepted causes of OSA include anatomical abnormalities of the upper airway and dysfunction of the upper airway dilator muscles. In a magnetic resonance imaging study (MRI), the thickness of the lateral pharyngeal muscle walls was found to be a significant factor associated with OSA^8^. Enlargement of the oropharyngeal soft tissue structures, particularly of the lateral pharyngeal walls, is associated with a risk of developing OSA. The dimensions of the nasopharynx, cross-sectional area at the hard palate level, and pharyngeal length are reportedly associated with OSA severity^9^. In a study investigating muscle activity, ventilation, and upper airway resistance during wakefulness and sleep onset in young healthy males, middle-aged healthy males, and middle-aged males with OSA, graded difference was noted in upper airway muscle activity during normal respiration among these groups. Notably, age and the presence of OSA affect the response of peripharyngeal muscles to inspiratory loading^10^. On the contrary, the role of genioglossus muscles in maintaining upper airway patency in OSA pathologies remains controversial^10,11^.

Aging, obesity, and male sex are the major risk factors for OSA^12^. While controlling BMI, the prevalence of sleep apnea increases but apnea severity decreases with age^13^. Pharyngeal anatomy progressively deteriorates with aging, and the related increase in upper airway dysfunction with advanced age likely contributes to increased upper airway collapsibility^14^. Regarding obesity, a longitudinal study found that 10% increase in weight can predict a 6-fold increase in the probability of developing moderate-to-severe sleep-disordered breathing^15^. However, the role of smoking in body mass index (BMI) should be considered. Smoking may help control body weight, and its quitting could induce weight gain, which may influence the risk of OSA given that BMI is one of the strongest risk factors for OSA. In several epidemiologic studies, higher prevalence of OSA in the male population has been confirmed^16,17^; however, a relatively lower awareness of OSA in females owing to its atypical symptoms might have led to its underdiagnosis in females^18^. Moreover, sex hormones possibly play protective roles in premenopausal females^19^. Sex-specific differences in terms of smoking behaviors and OSA etiology might be related to the result.

The role of smoking in OSA was unclear in previous studies. In the University of Wisconsin Sleep Cohort Study, heavy smokers were found to be at a greater risk of developing mild or moderate-to-worse sleep-disordered breathing^20^. On the other hand, a recent systematic review and meta-analysis of 14 identified studies with 5264 subjects found that the risk of OSA was not associated with cigarette smoking but was positively associated with alcohol consumption in cross-sectional or case–control studies^21^; the populations examined in these studies were White, African-American, and African-Indian. The available evidence fails to conclusively establish a clinically significant association between cigarette smoking and OSA^22^. Thus, the purpose of this cross-sectional study was to examine the association between cigarette smoking and OSA in a Taiwanese population cohort.

## METHODS

### Study population

In this retrospective chart review, we collected sleep-related data from the sleep laboratory charts of Lukang Christian Hospital between January 2012 and June 2013. We excluded subjects aged below 20 years. The study protocol was approved by the Institutional Review Board of Changhua Christian Hospital. No commercial funding was acquired for this study. In total, 733 Taiwanese subjects were recruited for this study.

### OSA assessment

The diagnosis of OSA usually requires overnight PSG or home sleep apnea testing to detect the frequency of apnea and hypopnea events. In this study, we used overnight PSG in a sleep laboratory for the diagnosis of OSA. The apnea–hypopnea index (AHI) measures the average number of disordered breathing events per hour; severity is classified as mild (AHI = 5–14), moderate (AHI = 15–30), and severe (AHI > 30). According to the International Classification of Sleep Disorders (3rd Edition), OSA is defined as AHI ≥5 with associated symptoms such as excessive daytime sleepiness; fatigue; impaired cognition; waking episodes with breath holding, gasping, or choking; and habitual snoring, breathing interruptions, or both during a patient’s sleep; or AHI ≥15 with/without associated symptoms. In this study, we defined OSA as AHI >15 (OSA group) and non-OSA as AHI ≤15 (control group).

### Smoking assessment

We collected data regarding sleep, current cigarette smoking status, sex, age, BMI, neck circumference, Epworth Sleepiness Scale score, and polysomnographic (PSG) sleep parameters. These data were collected by PSG study technicians at the PSG study.

### Statistical analysis

Demographic data are presented as mean and standard deviation values for continuous variables and as percentages for discrete variables. The prevalence of sex and smoking in each group was calculated, and 95% Clopper–Pearson confidence bounds were estimated. For univariate analysis of categorical variables, chi-squared and Fisher’s exact tests were used. Further, continuous variables were analyzed using Student’s t-test and one-way analysis of variance with post hoc corrections as required. Associations between OSA and other variables were calculated by logistic regression analysis. Statistical analyses were performed using the software program SPSS PC 17.0 (SPSS Inc., Chicago, IL, USA).

## RESULTS

Mean age of the study subjects was 46.99 ± 13.77 years; mean BMI and neck circumference were 25.97 ± 4.73 kg/m^2^ and 37.05 ± 13.25 cm, respectively. OSA was present in 215 subjects, of which 121 had severe OSA and 94 had moderate OSA. There were 151 smokers and 582 non-smokers in the cohort. The OSA group had significantly older age, higher BMI, and greater neck circumference than the control group. Moreover, the proportion of male subjects and smokers was significantly higher in the OSA group than in the control group. Further, the OSA group had significantly lower sleep time, total sleep time, REM sleep proportion, NREM stage 2 proportion (N2), and NREM stage 3 proportion (N3) than the control group. Moreover, the OSA group had significantly higher arousal index and snore times but lower oxygen saturation than the control group. Table 1 summarizes the results of the above analyses.

**Table 1:** Difference between OSA subjects and non-OSA subjects in age, body mass index, neck circumference, gender, cigarette smoking, and PSG parameters

	*Non-OSA subjects N=518 (Mean±SD)*	*OSA subjects N=215 (Mean±SD)*	*p*
Age (years)	45.73±13.91	50.00±12.99	0.000**
Body mass index (kg/m^2^)	24.87±4.29	28.59±4.73	0.000**
Neck circumference (cm)	35.55±3.78	40.78±23.67	0.002*
Male gender (%)	51.35	80.00	**0.000##**
Cigarette smoking (%)	18.53	25.58	**0.035#**
Epworth Sleepiness Scale	6.86±4.94	8.92±5.44	0.000**
**PSG parameters**			
Sleep period time (mins)	414.30±58.14	402.53±63.10	0.019*
Total sleep time (mins)	361.31±72.38	345.74±71.22	0.008*
Wake time after sleep onset (mins)	53.34±46.33	56.29±40.08	0.386
Sleep efficiency (%)	82.20±13.67	82.25±11.93	0.961
REM sleep onset latency (mins)	134.75±73.92	140.30±82.73	0.398
Sleep REM stage (%)	16.50±7.52	15.34±7.05	0.047*
Sleep N1 stage (%)	30.84±165.45	36.49±26.89	0.452
Sleep N2 stage (%)	51.47±24.25	40.37±21.41	0.000**
Sleep N3 stage (%)	10.29±9.91	7.92±8.96	0.003*
Arousal index	16.66±12.76	53.41±204.74	0.000**
PLM index	9.27±21.25	7.75±22.08	0.390
AHI (total sleep time)	4.29±4.44	38.42±19.59	0.000**
AHI (REM sleep time)	10.32±13.24	44.98±47.70	0.000**
AHI (NREM sleep time)	3.30±4.16	37.29±21.76	0.000**
Snore times	856.19±930.63	1407.09±909.02	0.000**
Oxygen saturation (%)	86.80±5.62	73.58±10.19	0.000**

PSG: polysomnography, OSA: obstructive sleep apnea, REM: rapid eye movement sleep, NREM: non-rapid eye movement sleep, PLM: periodic leg movement index, AHI: apnea– hypopnea index. Student’s t-test and one-way analysis of variance *p<0.05, **p<0.001. Chi-squared test #p<0.05, ##p<0.001.

The proportion of males was higher among smokers than among non-smokers. Notably, the smokers were significantly older, had higher BMI, and showed higher scores on the Epworth Sleepiness Scale than the non-smokers. PSG data indicated that smokers had significantly less total sleep time and percentage of NREM stage 3 than non-smokers. Moreover, smokers had significantly higher AHI values for total sleep time or non-rapid eye movement sleep than non-smokers. Finally, smokers had significantly longer snore times during sleep than non-smokers (Table 2). There was a significant association between cigarette smoking and OSA in the unadjusted model (OR=1.51, 95% CI: 1.04–2.21). However, no significant association was found between smoking and OSA after adjusting for sex, age, and BMI in the study sample (OR=1.02, 95% CI: 0.66–1.57) (Table 3). Sex and BMI led to the attenuation in the association.

**Table 2:** Difference between subjects with cigarette smoking and subjects without cigarette smoking in gender percentage, age, body mass index, neck circumference, Epworth Sleepiness Scale and sleep PSG parameter

	*Subjects with cigarette smoking N=151*	*Subjects without cigarette smoking N=582*	*p*
Male gender (%)	88.7	52.2	0.00**

**Table:** 

	*Mean±SD*	*Mean±SD*	
Age (years)	44.72±12.57	47.58±14.02	0.023*
Body mass index (kg/m^2^)	26.86±4.73	25.74±4.71	0.01*
Neck circumference (cm)	38.42±3.69	36.69±14.74	0.158
Epworth Sleepiness Scale	8.38±5.43	7.23±5.08	0.015*
Sleep period time (mins)	399.44±64.90	413.81±58.14	0.008*
Total sleep time (mins)	352.12±68.73	357.94±73.26	0.379
Wake after sleep onset (mins)	49.44±37.74	55.44±46.13	0.141
Sleep efficacy (%)	82.71±13.64	82.09±13.06	0.605
Sleep onset latency (mins)	16.45±23.63	16.82±20.92	0.851
REM onset latency (mins)	138.10±83.25	135.93±74.85	0.759
REM (%)	15.33±7.05	16.38±7.48	0.122
N1 (%)	28.93±23.01	33.43±156.49	0.725
N2 (%)	47.64±20.93	48.37±24.73	0.74
N3 (%)	7.70±9.16	10.09±9.78	0.007*
Arousal index (per hour)	27.65±20.69	27.39±125.81	0.979
Leg movement with arousal (per hour)	2.11±6.66	1.74±5.66	0.491
Period leg movement index	9.02±22.00	8.77±21.38	0.901
AHI (REM)	23.93±22.51	19.59±34.26	0.141
AHI (NREM)	18.56±23.24	11.89±18.53	0.000**
AHI (TST)	19.37±22.43	12.99±18.03	0.000**
Snore times	1379.03±1098.41	924.05±894.53	0.000**
O_2_ level	81.18±9.59	82.81±9.69	0.076

REM: rapid eye movement sleep, NREM: non-rapid eye movement sleep, AHI: apnea hypopnea index.

* p<0.05

** p<0.001

N1 – stage 1 NREM. N2 – stage 2 NREM. N3 – stage 3 NREM.

**Table 3:** Unadjusted (OR) and adjusted odds ratios (AOR) for the association between OSA and cigarette smoking

*Variables*	*Non-smoking (N=582 )*	*Smoking (N=151 )*	*OR ( 95% CI)*	*AOR ( 95% CI)^a^*
	*n%*			
OSA	160 (27.5)	55 (36.4)	1.51 (1.04–2.21) *	1.02 (0.66–1.57)

a Adjusted odds ratio for sex, age, and BMI.

* p<0.05.

OSA: obstructive sleep apnea, CI: confidence interval, BMI: body mass index.

## DISCUSSION

In this study, we found male sex, older age, greater neck circumference, and obesity (higher BMI) to be factors associated with OSA. Smokers demonstrated higher Epworth Sleepiness Scale scores, shorter sleep times, lower percentage of slow-wave (deep) sleep, higher AHI over their total sleep time or during non-rapid eye movement sleep, and longer snore times. These results represent poor sleep quality and excessive daytime sleepiness among smokers. Remarkably, after adjusting for sex, age, and BMI, we found no significant association between cigarette smoking and OSA.

There are several previous studies describing a strong association between cigarette smoking and OSA. Kashyap et al.^23^ found that current smokers are 2.5-fold more likely to have OSA than former smokers and non-smokers combined. According to the University of Wisconsin Sleep Cohort Study, current smokers were at a significantly greater risk of snoring (OR=2.29) and moderate-or-worse sleep-disordered breathing (OR=4.44) than subjects who never smoked20. In a study by Kim et al.^24^, moderate-to-severe OSA was more common among smokers and apnea, hypopnea, and oxygen desaturation indices were higher in this population.

The possible mechanisms underlying the described association between smoking and OSA include inflammation of the upper respiratory airways and impairment of the upper airway neuromuscular protective reflexes. Smoking possibly induces chronic inflammation of the upper respiratory airways by inducing cellular hyperplasia, edema, epithelium thickening, and/or ciliary dysfunction. A study including subjects who underwent uvulopalatopharyngoplasty found that the lamina propria of the uvula was thicker in smokers than in non-smokers and that there was a significant correlation between lamina propria thickness and OSA severity^24^. This result supports the association between cigarette smoking and OSA. Impairment of the upper airway neuromuscular protective reflexes induced by nicotine is another potential explanation for the effect of cigarette smoking on OSA. In the capsaicin aerosol test, cough sensitivity was found to be significantly diminished in a current smoker group compared with that in a control group^25^. Thus, decreased neuromuscular protective reflexes of the upper airway might worsen OSA.

However, other studies concluded that there is no association between cigarette smoking and OSA. In a study by Casasola et al.^26^, apnea index, AHI, and desaturation index did not significantly differ between smoking and non-smoking groups, but significant decreases were noted in nocturnal oxygen saturation among smokers. In a large community-based study comprising 6132 participants, previous exposure to smoking was associated with the worsening of OSA, although current smoking was not^27^. Further, AHI was lower among current smokers, suggesting that smoking is associated with lower prevalence of OSA. In another study, Conway et al.^28^ demonstrated that smoking habits are associated with arousal and oxyhemoglobin desaturation during sleep but not with AHI^28^. Using logistic regression analysis, they also noted that smoking status and pack-years are associated with AHI ≥5^28^. In another study, current smoking was found to be strongly and inversely associated with self-reported OSA diagnosis in men and women^29^. Nicotine from cigarettes may stimulate upper airway muscle and breathing and subsequently reduce airway collapsibility, and the susceptibility to OSA was supposed^30^.

To the best of our knowledge, this is the first relatively large-sample study to analyze the putative association between cigarette smoking and OSA in an Asian population. Our findings are consistent with those of previous studies demonstrating a lack of association between cigarette smoking and OSA^26-28^. Indeed, we noted that cigarette smoking was associated with less slow-wave sleep and higher Epworth Sleepiness Scale scores, which is consistent with the findings reported by Conway et al.^28^.

### Limitations

There are several limitations to this study that provide directions for future research. First, we did not collect data on cigarette smoking history, which would have included details such as smoking duration and daily smoking amount. Second, we did not collect data on family histories regarding medical illnesses and medications, which might correlate with OSA. Third, we used overnight PSG data for OSA diagnosis although two-night PSG data might facilitate higher diagnostic precision due to the elimination of the first-night effect^31^. Finally, the retrospective cross-sectional design is another limitation in itself; a prospective, controlled cohort study might provide stronger evidence.

## CONCLUSIONS

In this study, we highlighted poor sleep quality and excessive daytime sleepiness among cigarette smokers; however, we did not find any significant association between cigarette smoking and OSA after adjusting for age, sex, and BMI. Further well-designed, prospective controlled cohort studies might be helpful in clarifying the association between cigarette smoking and OSA.

## CONFLICTS OF INTEREST

Authors have completed and submitted the ICMJE Form for Disclosure of Potential Conflicts of Interest and none was reported.
